# On the Theory of Spasmo-Paralysis in Infants and Adults

**Published:** 1848

**Authors:** Marshall Hall

**Affiliations:** London


### ARTICLE IV.

*On the Theory of Spasmo-paralysis in Infants and Adults.* By MARSHALL HALL, M.D., F.R.S., &c., London.

Paralysis may depend on the exclusion of the influence either of the cerebrum; or the spinal marrow—that is, of both cerebrum and spinal marrow. Spasm can only arise from irritation of some part of the *spinal* system; but this irritation may effect the incident excitor nerves, the spinal centre, or the muscular nerves. Spasmo-paralysis is a term which I have adopted to express the varied combinations of spasm and paralysis, which occur so frequently in practice.

How shall we find our way through this labyrinth?

It may, indeed, be said that the diseases of the nervous system constitute one-third part of medicine, and that their accurate diagnosis was impossible, previous to the distinction of the three divisions of that system from each other. Now, this distinction depended, in its turn, on the detection and separation of one of these—the reflex spinal system—from the other two—viz.: the cerebral and ganglionic; and this again, in *its* turn, affords the diagnostic of the diseases of the entire nervous system.

Such is the important practical object to which I wish to devote myself, while I leave the mean and ignoble caviller at the threshold of this true temple of medical science.

Infants are often born with distortion of the foot or feet, and during growth a paralytic weakness and atrophy are conjoined with the spasmodic action of the deforming muscle. A similar effect is sometimes seen to take place during infancy. I have once seen a case in an adult in which the tendo Achillis became so drawn that the patient could no longer put the heel to the ground. In some cases of hemiplegia, spasmodic contraction of the hand and arm accompanies the paralytic attack. In other cases, a spasmodic contraction of the hand gradually takes place more remotely after the attack. I have seen various cases of paralysis, or spasm, distinctly, and of the two either variously combined or following each other. Now what is the just rationale—what the theory of these spasmo-paralytic cases?

These questions could not be answered before the appearance of the remarkable work of M. Flourens, or before the detection of the reflex spinal system. The due limitation of the excitor and *in*-excitor portions of the nervous system, which we owe to M. Flourens, and the just appreciation of the incident and reflex forms of action, of their excito-motor powers, which has flowed from my own investigations, are the essential preliminaries to an investigation of the theory of spasm, of paralysis, and of spasmo-paralysis. To this very day I observe these things confounded together, and that even by teachers of our pupils and critics of our literature.

*Intra-uterine Spasmo-paralysis.*

How interesting and how valuable would be a series of accurate cases and post-mortem examinations of the various congential spasmodic and spasmo-paralytic affections! of cheirismus, and especially of podrismus, in the varied forms, or rather deformities, of club-foot.

Is the cause of this calamity always of centric origin? or is it sometimes the reflex action of external cold, injury, &c?

The class of intra-uterine diseases still requires renewed investigation, and no part of it more than affections of the nervous system.

Effusion over the hemispheres, and at the base of the encephalon, and along the spinal canal, is too frequently the cause of irritation—pressure or counter-pressure—on the spinal system, that division of the nervous system endowed with the excito-motor power. This irritation is the source of various congenital, convulsive or spasmodic affections; it may be the cause of strabismus, laryngismus, &c, of the various distortions of the hands, and especially of the feet. In the case of two brothers, similarly affected, the tendo Achillis was permanently contracted with spasmo-paralysis of both legs; on the death of one, aged twelve, effusion on the cerebral hemispheres, at the base of the brain, and along the spinal canal, was found in considerable quantity; the arachnoid was thickened, and, over the lateral portion of one hemisphere, converted into a thin layer of bone.

### *Of Spasmo-paralysis in Infants and Children.*

Spasmo-paralysis in infants and children, is of centric and of ex-centric origin—the prognosis of the former being, of course, far more formidable than that of the latter.

Teething, and gastric, and intestinal irritation, and, I *suspect*, exposure of the naked surface to the cold, are the causes of the reflex or ex-centric forms of this malady. From such causes, I have seen hemiplegia of the arm, or of the leg, or of both; and the proof that the affection *was* of reflex origin was a very happy one—viz.: speedy recovery.

The event, however, is not always so fortunate.

Sometimes both legs are affected, and this affection is sometimes more observed in one leg than in the other; sometimes the spasm, sometimes the paralysis, predominates; and sometimes one leg is affected with paralysis, whilst the other is affected with spasmo-paralysis.

### *Spasmo-paralysis in the Adult.*

But of all the cases which have come under my observation, none has been more replete with interest and anxiety, than the spasmo-paralysis occurring in the adult period of human life.

It is well known that the epileptic convulsion sometimes leaves one arm, one leg, or one side, paralytic or hemiplegic, in a greater or less degree. If the seizures were not to be repeated, I imagine this paralysis would frequently subside, being the effect of shock, and of the common cause or causes of the convulsion and of the hemiplegia, which is, therefore, not permanent. But if the shock be repeated, the paralysis may be permanent, although the convulsion subside.

In one most interesting case, a lady, aged thirty-five, was siezed with violent convulsion of the left side of the face, and of the left arm, the leg being unaffected; when the convulsion ceased, the face and arm were left extremely, if not perfectly paralytic. A degree of amendment took place; but the convulsions returned, occupying the same seats as before, and, on ceasing, again left the face, arm, and hand, absolutely paralytic.

This lady once had phlegmasia dolens after parturition, and this leg again became swollen. But the cause of the attack of convulsions seemed to be discovered in the condition of the intestines; for these convulsions were relieved by purgative medicines, but were excited, if those medicines acted too violently.

From the paralysis left by this serious attack or repetition of attacks, the patient recovered completely—an additional proof that the affection had, like many cases of epileptic seizure, arisen from some cause ex-centric to the encephalon or spinal marrow. And how invaluable is this fact, in reference both to our prognosis and treatment!

Indeed, I may here observe, that spasmo-paralysis is in every respect a disease of less hopeless character than pure paralysis, inasmuch as the irritation of an organ is a less severe affection than its destruction. The diagnosis or detection of the cause is the first great object of the physician, and especially the determination of the question—whether that cause be seated centrically or ex-centrically.

In one case, which occurred in a member of our own profession, after repeated threatenings supposed to be apoplectic, severe spasmo-paralysis supervened, and remained permanent. Bleeding had been resorted to constantly as *the* preventive. It ought, I believe, to have been decided, but not too severe, antacid aperients, with a strict attention to the diet, which should not have been of a mere vegetable, but of a light and digestible character.

There was, I believe, more of the epiletic than of the apoplectic in those threatenings. Is there any physical lesion? Is the case, or was the case, one admitting of recovery? How deeply interesting are all these questions!

It is plain that the new topic—new because now viewed distinctly—of spasmo-paralysis, will assume an important position amongst the objects of the physician's studies.

I have two patients under my care, at this time, with podrismus, occurring at the ages, in one, of twenty-five, in the other, of forty-five. Both are females. In the first, the right foot is drawn upwards and inwards, and so severely as to induce great tenderness and swelling of the outer ankle. Various symptoms of nervous origin are conjoined with this deformity of the foot. In the other, the tendo Achillis in each leg is tense, and the toe only, and not the foot, much less the heel, can be put to the ground. In this case almost every article of food or medicine is rejected by vomiting.

I do not believe that either of these cases is hysteria. There is no other symptom of hysteric character, and the temperament in both patients is staid and sedate.

*Conclusion.*

From the recent progress of the physiology of the nervous system, we are now enabled to conclude—

1. That *paralysis*, pure paralysis, *may* be an affection either of the cerebrum, the spinal marrow, or the nerves ; but

2. That *spasm must* be an affection of some part of the true spinal system ; and

3. That spasmo-paralysis must at least involve in it an affection of the true spinal system, either primarily or secondarily.

There is only one exception to this last rule : it is the case of severe hemiplegia, in which, from the mere facts of the severing of the influence of volition, and the normal or physiological action of the spinal marrow—the source at once of the irritability of muscular fibre and of tone—the affected hand frequently becomes spasmodically flexed.

Here I conclude this brief paper. I think I have clearly shown in it, once more, how important, how essential, physiology is to the physician, and pointed out a distinction to be carefully drawn between paralysis, and spasm, and spasmo-paralysis, as at once a guide to our prognosis and our treatment.—*London Lancet.*

